# The important role of ferroptosis in inflammatory bowel disease

**DOI:** 10.3389/fmed.2024.1449037

**Published:** 2024-10-07

**Authors:** Hanhan Xie, Chun Cao, Dan Shu, Tong Liu, Tao Zhang

**Affiliations:** ^1^The Second Affiliated Hospital of Chengdu Medical College, China Nation Nuclear Corporation 416 Hospital, Chengdu, China; ^2^School of Biological Sciences and Technology, Chengdu Medical College, Chengdu, China; ^3^The Third Affiliated Hospital of Chengdu Medical College, Chengdu Pidu District People’s Hospital, Chengdu, China

**Keywords:** inflammatory bowel disease, intestinal epithelial cells, ferroptosis, ROS, mechanism

## Abstract

Ferroptosis is a type of regulated cell death that occurs due to the iron-dependent accumulation of lethal reactive oxygen species (ROS) from lipids. Ferroptosis is characterized by distinct morphological, biochemical, and genetic features that differentiate it from other regulated cell death (RCD) types, which include apoptosis, various necrosis types, and autophagy. Recent reports show that ferritin formation is correlated to many disorders, such as acute injury, infarction, inflammation, and cancer. Iron uptake disorders have also been associated with intestinal epithelial dysfunction, particularly inflammatory bowel disease (IBD). Studies of iron uptake disorders may provide new insights into the pathogenesis of IBD, thereby improving the efficacy of medical interventions. This review presents an overview of ferroptosis, elucidating its fundamental mechanisms and highlighting its significant involvement in IBD.

## 1 Introduction

Inflammatory bowel disease (IBD) is a chronic, incurable disorder that poses low mortality, stable course, and recurrence, and is a global health problem whose incidence and prevalence are steadily increasing (1). IBD manifests in two major forms, namely Crohn’s disease (CD) and ulcerative colitis (UC). These two forms are characterized as distinct chronic and recurrent conditions and exhibit different pathological features. The CD has the potential to impact different regions of the gastrointestinal tract. However, it is commonly observed that the terminal ileal or perianal region is more susceptible to CD, resulting in a discontinuous type of transmural inflammation. Furthermore, the CD is commonly correlated to complications, including abscesses, fistulas, and strictures. In contrast, UC is characterized by persistent inflammation of the mucosal and submucosal layers of the colon, with the initial manifestation of the disease typically occurring in the rectum and subsequently extending gradually along the colon. The histopathological features of UC include the presence of a large number of neutrophils in the lamina propria and crypt, where they form microabscesses. In contrast to UC, CD is characterized by the accumulation of macrophages, which often form non-tyrosine granulomas (1, 2). Although the etiology of IBD remains unclear, studies suggest that IBD pathogenesis may be attributed to immune response, external environment, individual genetics, intestinal microflora, and type of food intake (3).

Patients with IBD were found to have an altered intestinal microenvironment, often correlated to higher reactive oxygen species (ROS) levels, leading to damage to the intestinal epithelial barrier and inflammatory response (4). ROS can cause oxidative damage to lipid membranes by polyunsaturated fatty acids (PUFA), resulting in large amounts of lipid peroxides, which can lead to membrane damage and ferroptosis (ferroptosis) in intestinal epithelial cells (IECs) (4). IECs are a core component of the intestinal epithelial barrier. Moreover, abnormal death of IECs caused by ROS can lead to extensive epithelial erosion and disruption of the intestinal epithelial barrier, resulting in the development of UC (5, 6). In addition, ROS can act as messengers to regulate the polarization of intestinal macrophages; both NOX2-and NOX4-derived ROS can promote the polarization of primary macrophages into M1 (7) and M2 macrophages (8), respectively. The imbalance in polarization toward pro-inflammatory (M1) and anti-inflammatory (M2) phenotypes can lead to disturbances in intestinal immune homeostasis and inflammatory responses, which have a close association with IBD severity.

Recently, cell ferroptosis was found to be involved in the development of IBD. Excessive iron intake has been reported to exacerbate the colitis model triggered by dextran sodium sulfate (DSS) in rats and increase UC risk (9, 10). A study conducted by Xu et al. revealed that ferroptosis-related genes were highly expressed in human colon biopsy samples, including ACSL4 solute carrier family 38 member 1 (SLC38A1) and glucose-6-phosphate dehydrogenase (G6PD) (11). Collectively, these results indicate a probable link between IBD and ferroptosis, such as disruption of IECs and damage to the intestinal mucosal barrier, which ultimately results in the development of IBD. Ferroptosis is a type of regulated cell death (RCD) that occurs due to the iron-dependent accumulation of lipid ROS and is characterized by distinct morphological, biochemical, and genetic features that differentiate it from other RCD types, which include apoptosis, various necrosis types, and autophagy (12, 13). The iron poisoning process is primarily mediated by the accumulation of lethal ROS and lipid peroxidation products and is significantly affected by iron metabolism (14). Excess iron resulted in ROS formation through the Fenton reaction (14, 15). Following this, a significant amount of ROS results in glutathione peroxidase 4 (GPX4) deactivation via glutathione (GSH) degradation (16). Furthermore, ROS interact with PUFAs present in cellular lipid membranes, resulting in producing numerous lipid peroxides that impair the integrity of cell membranes and ultimately result in cell death (17). Ferroptosis can occur in various disorders, including acute injury, myocardial infarction, ischemia/reperfusion (I/R), and cancer (1, 4, 7). Ferroptosis was revealed to be involved in the two major forms of IBD: CD and UC (18–21). In addition, inhibition of ferritinase by small molecule compounds alleviates experimental colitis triggered by DSS (22, 23). To conclude, ferroptosis appears to have significant implications in intestinal disorders and could potentially offer novel avenues for the management of IBD. The present review offers a comprehensive depiction of ferritin formation and its significant involvement in IBD.

## 2 Ferroptosis: a unique form of regulated cell death

### 2.1 Definition, characteristics, and development of ferroptosis

The concept of ferroptosis can be traced back to the discovery of erastin, a new agent identified in 2003 as an ferroptosis inducer. This was accomplished by screening a library of small molecule drugs that exhibited selective lethality in RAS-mutated cancer cell lines, thereby introducing a new non-apoptotic mechanism of cell death (24). Subsequently, Yagoda et al. identified the direct target of erastin to be mitochondrial voltage-dependent anion channel 2 (VDAC2, 25). Moreover, Yang et al. identified two compounds, RAS-selective lethal 3/5 (RSL3/5), with similar properties to erastin (25). They found that iron chelators have the ability to inhibit the cell death process. The nomenclature “iron poisoning” was established by Dixon et al. in 2012 to describe this newly discovered mechanism of cell death (13). This process was formally named ferroptosis, which is characterized by being iron-dependent, non-apoptotic, as well as having a large accumulation of lethal lipid ROS. This pattern is different from other types of RCD that have been previously observed. From a morphological point of view, iron-intoxicated cells exhibit certain characteristics such as small mitochondria, condensed mitochondrial membrane density, reduced or absent mitochondrial cristae, and ruptured outer mitochondrial membranes (13, 26). From a biochemical point of view, intracellular iron and ROS increase, leading to GSH depletion and impaired GPX4 activity. Consequently, this results in a breakdown of lipid peroxide metabolism, causing significant quantities of lipid ROS to accumulate and ultimately fostering ferroptosis (27). From a genetic point of view, ferroptosis is a complex biological process correlated to metabolic disturbances in several biological processes, including iron homeostasis, fatty acid synthesis, and lipid oxidation, which are regulated by an organized network of genes (28). Ferroptosis can be triggered by multiple substances, such as erastin, RSL3, p53, DPI7, FIN56, and FINO2, among others. These molecules trigger ferroptosis by different methods, GSH depletion, GPX4 inactivation, or degradation (13, 29). As the mechanism of ferroptosis has been studied, many specific inhibitors of ferroptosis have been identified, including ferrostatin-1(Fer-1), liproxstatin-1, and vitamin E, as well as iron chelators, DFO, and deoxynivalenol methanesulfonate. These substances suppress ferroptosis through the inhibition of lipid peroxide formation and iron accumulation. In addition, ferroptosis is positively or negatively regulated by several transcription factors, including nuclear factor E2-related factor 2 (NRF2), activating transcription factor 3/4 (ATF3/4), Yes-associated protein 1 (YAP1), and hypoxia-inducible factor lα (HIF1A) (28).

### 2.2 Mechanism of ferroptosis

**Figure 1:**
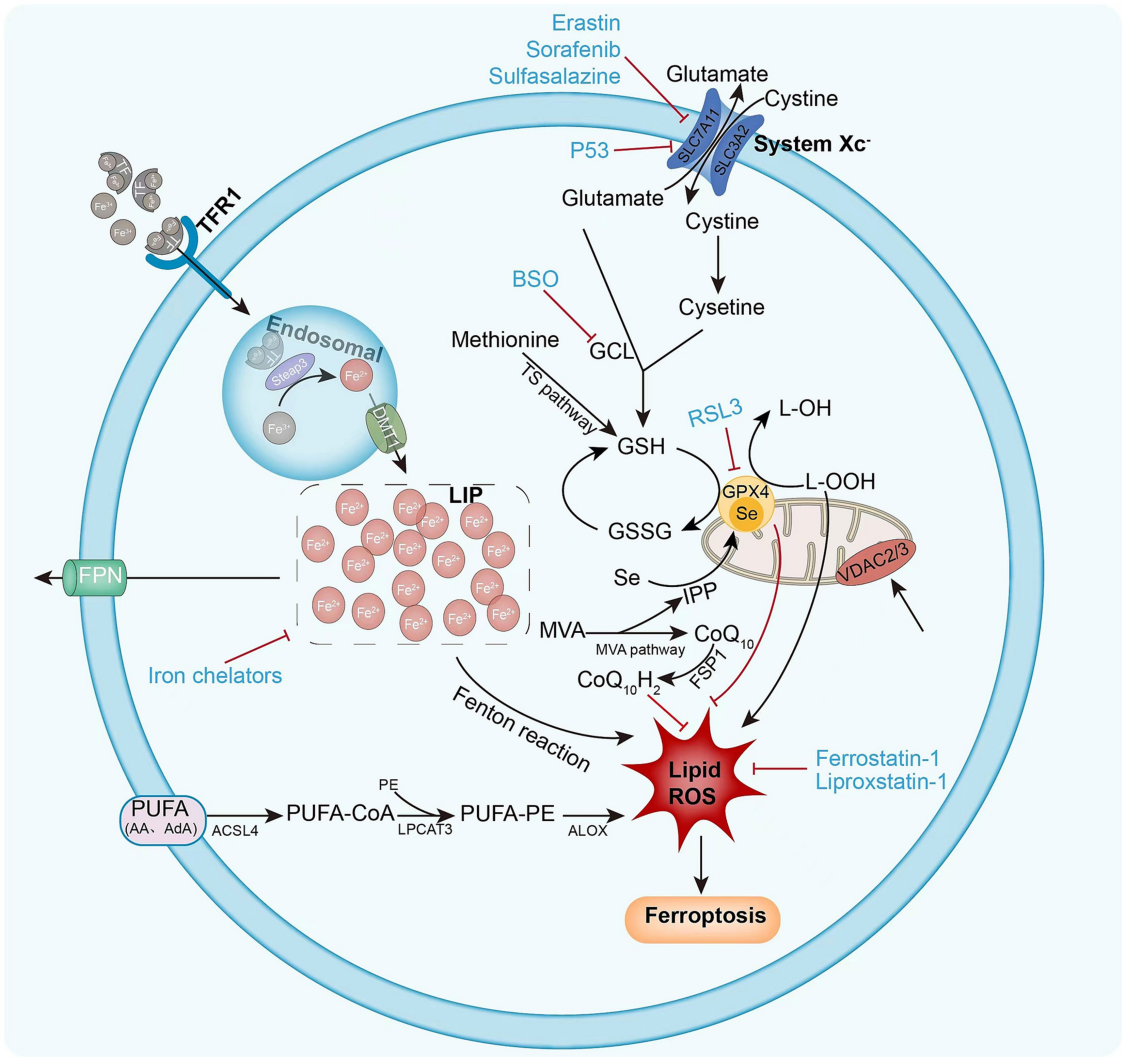
Mechanism of intracellular ferroptosis. Ferroptosis is defined by iron accumulation, excessive ROS production, and massive lipid peroxidation. Ferroptosis initiation and execution involves three major metabolic pathways: the amino acid/GSH, lipid, and iron pathways. Ferroptosis sensitivity is also controlled via additional signaling pathways and regulators. The following diagram illustrates the ferroptosis process and provides an overview of the key molecules and targets that govern iron and lipid peroxidation regulation. ACSL4 (acyl-CoA synthase long-chain family member 4), BSO (buthionine sulphoximine), DMT1 (divalent metal transporter 1), FSP1 (ferroptosis suppressor protein 1), FPN1 (ferroportin 1) GPX4 (glutathione peroxidase 4) GSH (glutathione), GSSG (oxidized glutathione), GSS (glutathione synthetase), GCL (glutamate-cysteine ligase), LOX (lipoxygenase), LPCAT3 (lysophosphatidylcholine acyltransferase 3), PUFA (polyunsaturated fatty acid), PE (phosphatidylethanolamine), ROS (reactive oxygen species), RSL3 (Ras-selective lethal 3), STEAP3 (six-transmembrane epithelial antigen of prostate 3 metalloreductase), SLC7A11 (solute carrier family 7 member 11), TF (transferrin), TFR1 (transferrin receptor 1), VDAC2/3 (voltage dependent-anion channel 2/3).

The sources of lipid ROS that induce ferroptosis are multifaceted, and lipid peroxidation pathways and iron metabolism are increasingly considered to be involved in the ferroptosis process (14) (Figure 1).

#### 2.2.1 Iron metabolism in ferroptosis

**Figure 2:**
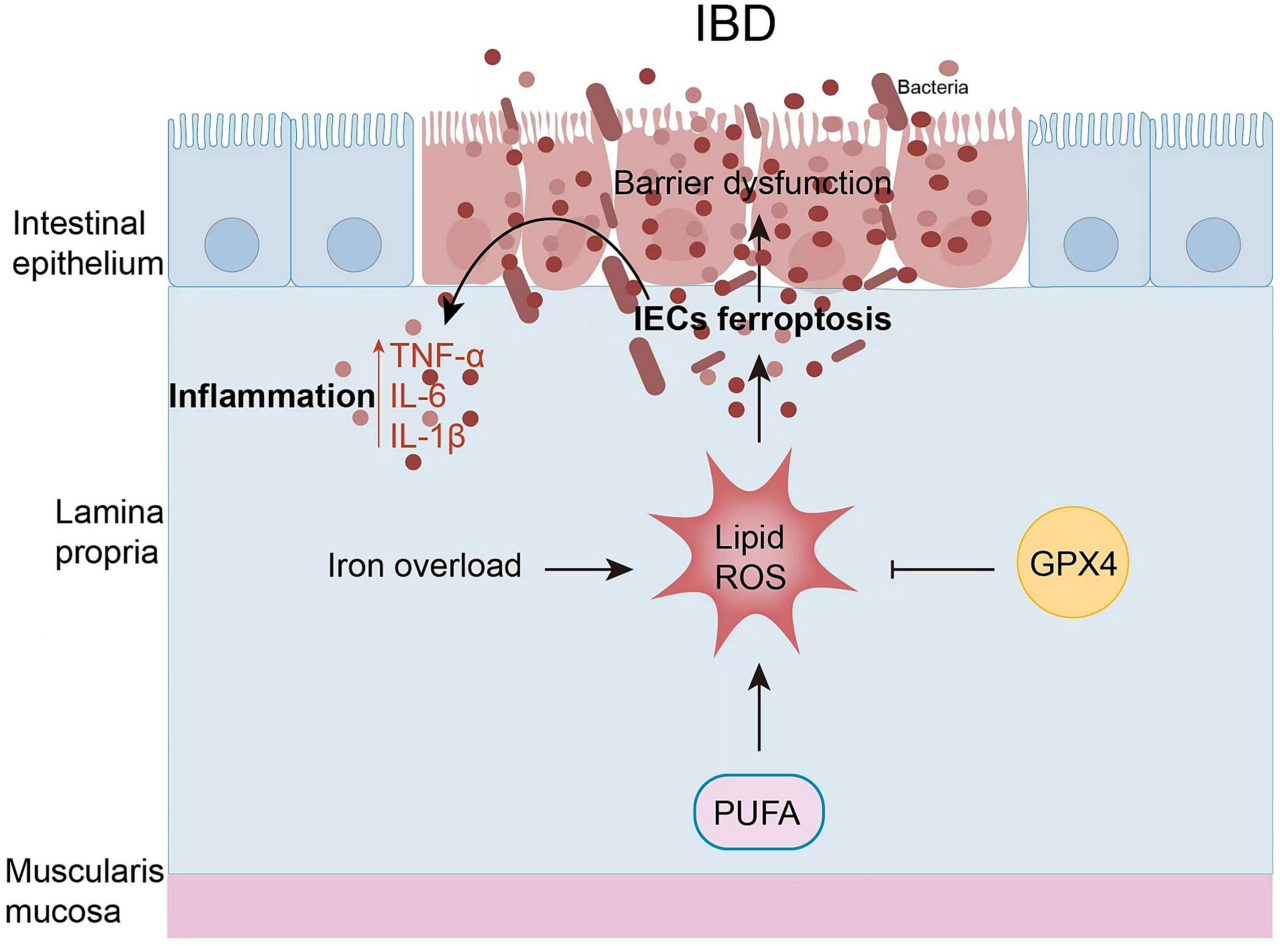
Iron metabolism in ferroptosis. Enterocytes absorb iron from the diet through the combined action of iron reductase (e.g., Dcytb) and the divalent metal transporter DMT1. The iron absorbed by the enterocytes is exported to the bloodstream via the ferroportin (FPN-1). At the same time, Fe^2+^ is oxidized to Fe^3+^ by ceruloplasmin. Subsequently, Fe^3+^ is loaded onto the circulating TF in the plasma.

The cellular metabolism is significantly influenced by the presence of iron, owing to its ability to transition between oxidized and reduced states. This transition facilitates the generation of ROS through a Fenton-like reaction, which ultimately results in nonenzymatic lipid peroxidation (30–32). Iron serves the dual purpose of promoting nonenzymatic reactions and being conveyed to iron-containing enzymes (lipoxygenases, 15-LOX), which are implicated in lipid peroxidation (32). Consequently, iron is required as a key factor in the formation of lipid peroxides for ferroptosis to occur. Normally, heme and non-heme iron are predominantly found in iron form (Fe^3+^). Fe^3+^ is reduced to Fe^2+^ in the intestinal lumen by iron-reducing enzymes, including duodenal cytochrome b reductase (Dcytb), and transported to the enterocytes via the divalent metal 1 transporter (DMT1). Subsequently, Fe^2+^ exported by ferroportin (FPN-1) can be oxidized to Fe^3+^ by membrane-bound ceruloplasmin, which in turn binds to transferrin (TF) to form TF-Fe^3+^, which is referred to as holotransferrin (transferrin bound to two iron atoms; Figure 2). Holotransferrin further binds to the membrane protein transferrin receptor (TFRC) to form a complex that is subsequently subjected to endocytosis (26, 31). Fe^3+^ is then reduced to Fe^2+^ by the endosomal iron reductase hexa-transmembrane prostatic antigen 3 (STEAP3) and transported across the endosomal membrane into the cytoplasm via DMT. Fe^2+^ is stored in the unstable iron pool (LIP), and ferritin is the major intracellular iron storage protein, which consists of ferritin light chain polymer (FTL) and FTH1. Excess intracellular Fe^2+^ is oxidized to Fe^3+^ and exported by FPN-1 (33). Cellular iron homeostasis is tightly regulated; in the presence of iron overload, such as reduced iron stores or increased iron uptake, excess iron can trigger ferroptosis. Knockdown of TFRC inhibits erastin-induced ferroptosis (34). Moreover, heme oxygenase 1 (HO-1) overexpression in HT-1080 fibrosarcoma cells exacerbates ergocalin-triggered cell death (35). However, silencing HO-1 increases the growth inhibition of hepatocellular carcinoma (HCC) cells after ferroptosis triggering, suggesting that HO-1 also has an anti-ferroptosis effect (36). The dual role of HO-in ferroptosis may depend on its expression and cell type. Nuclear receptor cofactor 4 (NCOA4) is a selective cargo receptor for ferritin phagocytosis, i.e., catabolism of ferritin by autophagy, and overexpression of NCOA4 increases ferritin catabolism and promotes ferroptosis (37). In addition, prominin 2 promotes ferroptosis in breast cancer through iron export mediated by the Prominin2-MVB/exosomal ferritin pathway (38). Other regulators, including iron response element binding protein 2 (IREB2) and heat shock protein beta-1 (HSPB1), may also regulate ferroptosis by mediating iron uptake (39, 40). Collectively, these findings indicate that iron is an indispensable component of ferroptosis.

#### 2.2.2 Lipid peroxidation pathway and iron reduction

Lipid peroxidation is a hallmark of ferroptosis, and PUFAs are one of the major targets of lipid peroxidation. ROS can attack PUFAs in lipid membranes, leading to lipid peroxidation. Free PUFAs (mainly arachidonic acid, AA) must be esterified to membrane phospholipids (PLs) by acyl-CoA synthase long-chain family member 4 (ACSL4) and lysophosphatidylcholine acyltransferase 3(LPCAT3). ACSL4 acylates AA, LPCAT3 catalyzes the acylated AA into membrane PLs, and then lipoxygenases (LOXs) catalyze PUFA PLs, producing native lipid peroxidation (41) (Figure 1). Several studies have identified ACSL4 and LPCAT3 as key determinants of susceptibility to ferroptosis, especially ACS4, which is considered a biomarker of ferroptosis (42). When GPX4 is inactive, AA and other PUFAs play an important role in ferroptosis, whereas ACSL4 and LPCAT3 promote RSL3-induced ferroptosis but not erastin-induced ferroptosis in KBM7 cells (43). ACSL4 sensitizes breast cancer cells to ferroptosis, whereas pharmacological ACSL4 inhibition prevents ferroptosis. Other researchers have reported that LPCAT3 deficiency can lead to resistance of fibroblasts to ferroptosis (42). This suggests that ACSL4 and LPCAT3 are key players in ferroptosis. It was shown that phosphatidylethanolamines (PEs) containing PUFA, especially those containing AA and epinephrine (AdA), are most susceptible to peroxidation in ferroptosis (44). Thus, overwhelming lipid peroxidation by PUFA leads to an increased susceptibility to ferroptosis.

Ferroptosis primarily results from an excessive amount of lipid peroxidation caused by the accumulation of ROS, which is reliant on the presence of iron, as previously noted. Ferroptosis can occur due to the impairment of GSH-dependent lipid peroxidation repair system, in addition to PUFA-mediated signaling. The proper functioning of GPX4, a member of the GPX family responsible for converting cytotoxic lipid hydroperoxides (L-OOH) into nontoxic lipid alcohols (L-OH), thus preventing the formation of lethal lipid ROS, is dependent on the indispensable cofactor GSH (45). GSH, an antioxidant, is a tripeptide consisting of glutamate, cysteine, and glycine. It serves as the rate-limiting enzyme for the *de novo* synthesis of GSH, which is promoted by glutamate cysteine ligase (GCL) and glutathione synthetase (GSS). Cysteine is regarded as the limiting factor in the production of GSH among these three amino acids. The system xc-antibody is a heterodimeric protein complex composed of the solute carrier family 7 member 11 (SLC7A11/xCT) and the solute carrier family 3 member 2 (SLC3A2) located on the plasma membrane. Its primary function is to facilitate the transmembrane transport of extracellular cysteine and the export of intracellular glutamate, which is subsequently converted to cysteine and synthesized by GSH (26). The depletion of GSH results in elevated levels of oxidative stress, molecular impairment, and consequent cell ferroptosis (46). Erastin and other molecules (e.g., salazosulfapyridine, sorafenib) can induce ferroptosis by inhibiting system x and reducing GSH production (13, 30). Additionally, it was shown that P53, a significant gene in tumor suppression, has the ability to reduce SLC7A11 expression to inhibit cystine uptake by system x_c_^−^, thereby impairing the activity of GPX4 and leading to decreased antioxidant capacity, ROS accumulation, and ferroptosis (47). The lack of SLC7A11 in mice leads to a significant increase in plasma cysteine concentrations, and the survival of embryonic fibroblasts derived from these mice is compromised. Antioxidants such as *β*-mercaptoethanol (2ME), N-acetylcysteine (NAC), or vitamin E prevent cell death (48). In the absence of SLC7A11, iron treatment may lead to ferroptosis in hepatocytes (49). The deletion of SLC7A11 by CRISPR-Cas9 induces ferroptosis in cultured PDAC cells (50). The known GCI inhibitor buthionine sulfonamide (BSO) can also reduce GSH synthesis, leading to ferroptosis (17). In conclusion, GSH depletion is involved to a great extent in triggering ferroptosis.

GPX4 is a crucial factor and a master regulator in ferroptosis. The suppression of GPX4 activity may result in ferroptosis by forming lipid peroxides. The inactivation of GPX4 can be achieved through both indirect approaches, including GSH depletion, as well as direct methods involving pharmacological or genetic interventions. RSL3 is able to induce ferroptosis by directly inhibiting the enzymatic activity of GPX4 through covalent interaction with selenocysteine at the active site of GPX4 (51). In addition to RSL3, other synthetic small molecules, including DPI7, DPI, JKE1674, and JKE-1716, can inhibit GPX4 activation (26). Like RSL3, these compounds induce ferroptosis by covalently interacting with GPX4 active site without depleting cellular GSH. These compounds are categorized as “class 2” ferroptosis-inducing compounds (FINs). in contrast to “class l” FINs, which include erastin and other system x inhibitors that have an indirect effect on the blockage of GPX4 activity by blocking GSH synthesis (16). Deletion of GPX4 in mice can lead to acute renal failure and ferroptosis. Lipophilic antioxidants (e.g., liproxstatin-1) inhibit ferroptosis in GPX4-deficient mouse cells (27). GPX4 activity requires selenocysteine, the synthesis of which is mediated by selenocysteine-tRNA. The maturation of selenocysteine-tRNA can be regulated by the pentadienoic acid (MVA) pathway, which in turn can impact the synthesis of GPX4 and the development of ferroptosis (52). Thus, inhibition of the MVA pathway may lead to ferroptosis, thereby validating the crucial role of GPX4 activity in preventing ferroptosis.

#### 2.2.3 Other mechanisms of ferroptosis

Ferroptosis can be modulated by alternative pathways as well. Mitochondrial VDACs are membrane-spanning channels that facilitate the transmembrane transport of ions and metabolites. Erastin can interact with VDAC2 protein to trigger mitochondrial malfunction and oxidative substance release, eventually leading to oxidative cell death, i.e., ferroptosis (53) (Figure 1). GSH can be synthesized via the sulfotransferase pathway, in which methionine is converted to cystine (54). Therefore, reduced methionine intake can lead to ferroptosis. Furthermore, the FSP1-CoQ10-NAD (P) H metabolic pathway (55) and p62-Keap1-NRF2 (36) exhibit proficient regulation of ROS formation of intracellular lipids and serve as a regulator of ferroptosis.

#### 2.2.4 Box one measuring ferroptosis

Cell ferroptosis can be detected by changes in cell morphology, cell viability, ROS iron content, lipid peroxidation levels, and gene expression associated with ferroptosis (Table 1). The utilization of transmission electron microscopy is a viable approach for the identification of distinct morphological characteristics of cellular ferroptosis (56). Cell viability can be measured with the Cell Counting Kit-8 assay (57). In addition, when ferroptosis occurs, the iron level of cells increases. We can test iron levels with the Iron Assay Kit or the Phen Green SK (PGSK) fluorescent probe, a cell membrane permeable dye employed to assess intracellular iron levels by flow cytometry or confocal microscopy (14, 36). ROS and lipid peroxidation measurements are important to support the development of ferroptosis. Cellular ROS levels can be measured with ROS detection kits and dihydroethidium (DHE) kits, which indirectly reduce ROS levels by measuring superoxide anions using fluorescence microscopy (22). Oxidative lipidomics is a powerful technique to study lipid oxidation, and other methods to detect lipid peroxidation include the use of C11-BODIPY or Liperfluo probes by fluorescence microscopy (58). In addition, levels of malondialdehyde (MDA) and 4-hydroxynonenal (4-HNE), byproducts of lipid peroxidation, may indicate cellular ferroptosis (59). Moreover, we were able to detect changes in gene/proteinexpression correlated with ferroptosis, including prostaglandin endoxylase 2 (PTGS 2), ACSL4, GPX4, and ferritin heavy chain 1 (FTH1) (4). In recent years, the detection of ferroptosis has risen from the cellular level to the genetic level with the advent of various high-throughput assays. The use of single cell RNA-sequencing technology to detect the enrichment of ferroptosis-related genes has led to more rapid and accurate detection of ferroptosis.

**Table 1:** Methods for detecting ferroptosis.

Detection object	Methods
The specific morphological features of ferroptosis	Transmission electron microscopy
Cell viability	Cell Counting Kit-8 assay (CCK-8)
Cellular iron levels	Iron assay kit; Phen Green SK (PGSK) probe
Cellular ROS content	ROS kit; DHE kit
Lipid oxidation	C11-BODIPY; Liperfluo probe
The by-products of lipid peroxidation	MDA kit; 4-HNE kit
Ferroptosis-related RNA	Single cell RNA-sequencing

## 3 Ferroptosis in IBD

### 3.1 Inflammation and ferroptosis

Inflammation is a pathologic self-defense process that eliminates destructive factors, removes necrotic tissue, and repairs damage caused by the body after being stimulated by various injuries. If the inflammatory response persists, it can contribute to the development of disease through various pathways. There is a close relationship between ferroptosis and inflammation: on the one hand, ferroptosis can enhance the inflammatory response through various pathways, leading to increased infiltration of inflammatory cells and inflammatory mediators; on the other hand, the activation of inflammation can trigger ferroptosis. It has been shown that pathogenic bacteria or viruses in inflamed tissues promote ferroptosis in cells while activating immune cells and triggering inflammatory responses through the action of lipopolysaccharides, peptidoglycans, bacterial or viral DNA, and host cell toll-like receptors carried or released by themselves (60). The increase in AA-induced by various factors and the activation of inflammatory cells promote ferroptosis by impairing mitochondrial function and generating large amounts of free radicals. In macrophages, the upregulation of 15-LOX expression catalyzes AA dioxide, leading to lipid peroxidation, which triggers ferroptosis in macrophages (51). A significant increase in serum levels of the inflammatory factors IL-1B, TNF-a, and IL-6 was found in hemoglobin-injected mice, whereas the use of inflammatory vesicle inhibitors significantly reduced the levels of hemoglobin-induced inflammatory factors. They also reduced ferroptosis, suggesting that hemoglobin induces inflammatory vesicle formation, which in turn leads to ferroptosis (61). The inflammation-related damage factors released by cells after ferroptosis activate immune cells that further promote the progression of inflammation by impairing AA-mediated lipid metabolism, decreasing mitochondrial function, increasing oxidative stress, and reducing GPX4.

### 3.2 Ferroptosis and IBD in the intestinal epithelium

**Figure 3:**
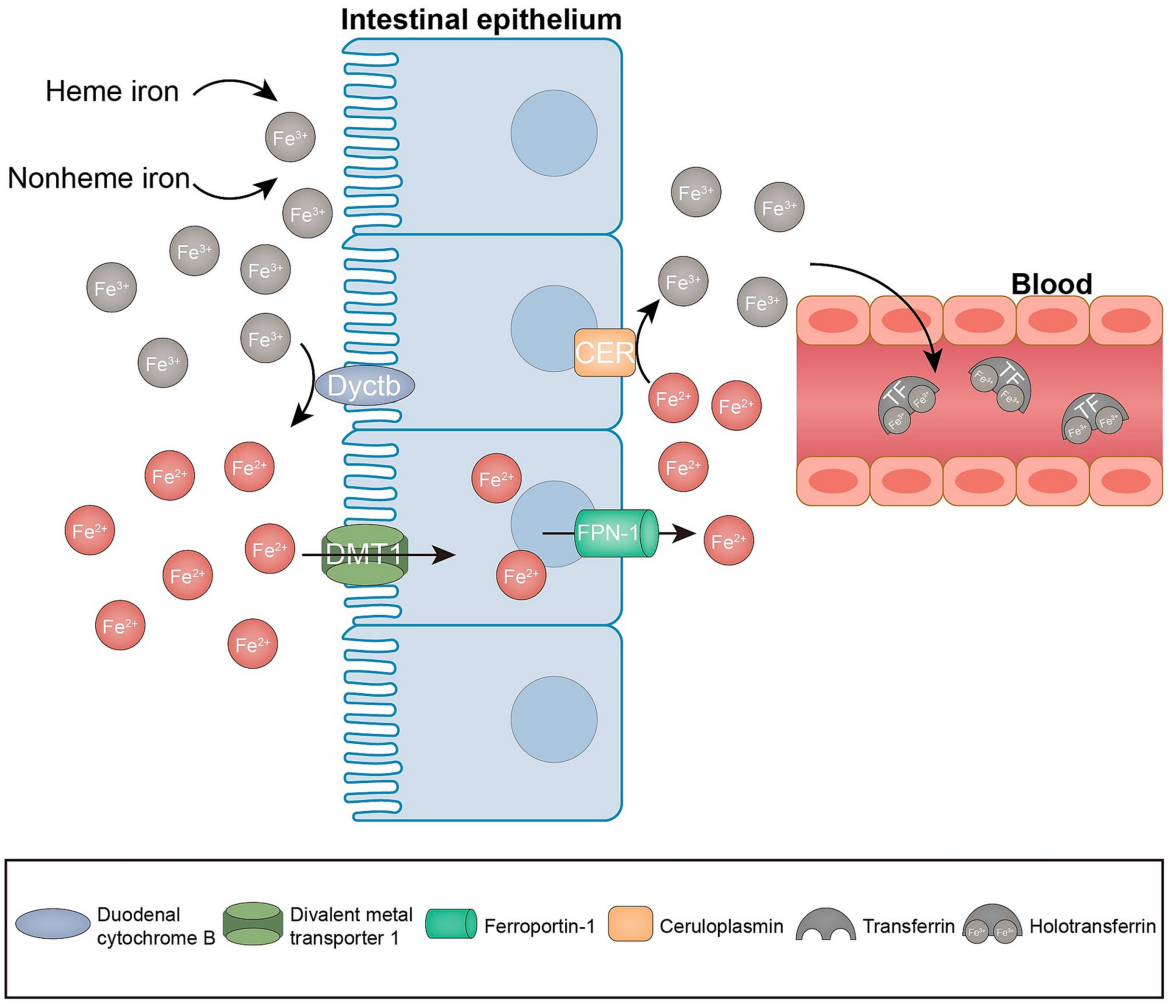
Ferritinosis in IBD. In recent studies, iron uptake disorders have been directly linked to the pathogenesis of IBD. IEC ferroptosis promotes the disruption of epithelial barrier function. As a result, immune cell and cytokine production is hyperactivated, ultimately leading to intestinal inflammation and epithelial damage.

The intestinal epithelium is the first line of defense for the intestinal mucosa. It consists of a single layer of tightly connected columnar epithelial cells that keep out foreign antigens, microorganisms, and their toxins (62). The maintenance and continuous renewal of IEC function is maintained by cell death of IECs, including apoptosis, necrosis, necrotizing, pyrotizing, and ferrotizing. However, the imbalance in cell death of IECs leads to increased intestinal permeability and disruption of barrier function, disrupting the balance of IECs, which in turn leads to IBD. There is increasing evidence that increased intestinal permeability may be associated with ferroptosis of IECs in IBD (Figure 3). In both patients with clinical UC and experimental colitis triggered by DSS in miceferroptosis-related genes were significantly up-and down-regulated, and cellular MDA content FTH and FTL protein levels were increased in IECs, and mitochondrial atrophy and mitochondrial cristae were reduced in colonic IECs of DSS-treated mice (11). The same was observed in clinical CD patients and in trinitrobenzene sulfonic acid (TNBS)-induced experimental colitis: increased expression of ACSL4/MDA/PTGS2/iron and inhibition of GPX4, which was ameliorated by treatment with Ferr-1 (63). Overall, these findings suggest that ferroptosis occurs in IECs with IBD and leads to increased inflammation.

### 3.3 Intestinal fat ferroptosis and IBD

Studies have shown that high-fat diets (HFD) are associated with an increased risk of IBD, especially diets rich in cholesterol and animal fats, such as polyunsaturated omega-6 fatty acids (PUFA) and arachidonic acid (AA) (64). The PUFAs have been observed to hinder the normal functioning of cells by creating cytotoxic protein adducts or by disrupting cell membranes through lipid peroxidation, leading to ferroptosis in IECs. GPX4 not only protects against lipid peroxidation (LPO) but also induces ferroptosis in cells either by the deletion of GPX4 allele in mice or its pharmacological inhibition. Mayr et al. found that GPX4 activity in CD epithelial cells was reduced, characteristic of LPO, and that PUFAs (especially AA) induced the release of interleukin 6 (IL-6) and chemokine (C-X-C motif) ligand 1 (CXCL1) from these lECs. Mice lacking the Gpx4 allele in IECs show signs of epithelial LPO and focal neutrophilic enteritis with a granuloma-like accumulation of inflammatory cells when fed a PUFA-rich diet (65).

Thus, a low-fat, high-fiber diet may reduce inflammatory factors and dysbiosis of intestinal flora in fetal samples and improve the life quality of patients with UC (66). Meanwhile, Zhang et al. reported that an HFD exacerbated colitis-associated carcinogenesis (CAC) by bypassing the ER stress-mediated ferroptosis pathway (67).

### 3.4 Dietary iron intake and IBD

Iron deficiency anemia is a prevalent complication in patients suffering from IBD, and iron replacement therapy (IRT) can improve anemia, including oral and intravenous iron supplementation, but iron overload can lead to ferroptosis (68). Iron is an indispensable element for the development of ferroptosis. Iron supplementation has been reported to alter the balance of intestinal microbiome and exacerbate CD-like intestinal inflammation in mouse models (69). Iron supplementation was found to exacerbate disease activity and oxidative stress in a rat model of colitis induced by DSS (10). In Japan, the incidence of UC has increased, and high iron intake has an effect on the development of UC compared to high zinc intake (9). Additionally, it is recognized that iron chelators have the capacity to decrease iron synthesis and ameliorate the colonic symptoms of IBD. Collectively, these results propose a potential association between IBD and the consumption of dietary iron.

### 3.5 Regulatory mechanisms of ferroptosis in IBD

Indeed, the molecular mechanisms of ferroptosis are diverse, and several transcription factors (e.g., TP53, NFE2L2/NRF2, ATF3, ATF4, YAPTAZTFAP2CSPHIFAEPAS/HIFA BACH TFEBJUNHIC1, and HNF4A) play multiple roles through transcription-dependent or transcription-independent mechanisms play multiple roles in shaping ferroptosis susceptibility (28). NRF2 has been identified as a potentially valuable therapeutic target for the management of IBD. The transcription factor, nuclear factor erythroid 2-like 2 (NRF2), governs the expression of antioxidant response elements, including GPX4 (70). Recently, Nrf2-Gpx4 pathway activation was found to have the ability to significantly suppress ferritin formation (71). Fer-1 has also been shown to alleviate DSS-induced colitis through the NRF2/HO-1 pathway (72). The small molecule inhibitor of Keap-Nrf2 protein–protein interaction, CPUY192018, has been found to exhibit cytoprotective properties against DSS in both NCM460 cells and mouse colon. This effect is achieved through the activation of Nrf2 signaling (73). Recent findings suggest that endoplasmic reticulum (ER) stress signaling is closely correlated to ferritin formation, which is thought to be a promoter of UC. Furthermore, phosphorylation of NF-кBp65 acts as an upstream regulator to inhibit ER stress-mediated IEC ferroptosis, thereby alleviating the disease (11).

## 4 Therapeutic strategies for ferroptosis

IBD has emerged as a pressing global health concern, which is anticipated to result in a substantial upsurge in healthcare expenditures due to its rapid increase in incidence and prevalence. Ferroptosis has recently been identified as a different type of RCD. As previously described, the fundamental characteristics of ferroptosis include iron overload, GSH depletion, GPX4 inactivation, and lipid peroxidation. The results of direct and indirect studies revealed a strong correlation between iron dystrophy and intestinal disorders. The clinical symptoms of IBD were revealed to include iron deficiency and anemia resulting from bleeding and malabsorption, which can significantly harm the well-being of affected individuals (74). Oral iron has been used in the clinical management of iron deficiency anemia in patients suffering from IBD (75). Nevertheless, an excessive amount of iron may cause iron overload in the gut, leading to the dysregulation of ROS production and disturbance of the gut microbiota. These effects could potentially worsen the condition of IBD (69). According to a study conducted on rats, the prevention of experimental colitis and gastric ulcers was observed through the oral administration of iron chelators deferiprone (DFP) and DFO (76). The enzymatic activity of GPX4 involves the elimination of lipid hydroperoxides and the prevention of ferroptosis. This function is attributed to its antioxidant properties. A decrease in GPX4 activity was observed in the IECs of CD patients as lipid peroxidation increased. - The consumption of diets that are high in PUFAs but low in saturated fatty acids has been found to induce focal enteritis in IEC-specific Gpx4 mice. +-More strikingly, the findings suggest that GPX4 plays a crucial role in preserving intestinal homeostasis by protecting against lipid peroxidation, as evidenced by the increased vulnerability of IEC Gpx4 mice to DSS-induced colonic inflammation relative to their wild-type counterparts (65). Curculigoside, a naturally occurring compound present in *Curculigo orchioides* Gaertn, has been discovered to possess diverse biological properties and has been shown to mitigate DSS-induced UC in mice. Curculigoside has been found to provide support for GPX4 expression, thus protecting against ferroptosis in a manner that depends on selenium (22). The efficacy of Astragalus polysaccharide (APS), a bioactive constituent of Astragalus membranaceus, in ameliorating experimental colitis has been demonstrated.

Together, these studies form the pathological concept of the involvement of ferric IECs in colitis. The presence of ferric IECs has been observed to cause a disturbance in the intestinal barrier, leading to the release of intestinal microorganisms and an overactive immune response within the intestines. This, in turn, has been linked to the worsening of mucosal lesions related to colitis. In addition, ferric IECs release a number of immunogenic molecules that potentially enhance the occurrence of local inflammation (77). However, it is unclear whether other intestinal immune cells besides IECs are ferroptosis in the pathogenesis of intestinal injury. Further investigation is necessary to determine whether the ferroptosis of specific types of immune cells in the intestine plays a role in the development or advancement of intestinal diseases. Given the beneficial effects of various ferroptosis inhibitors on ameliorating tissue damage associated with colitis (Table 2), there is significant therapeutic potential for targeting ferroptosis in colitis prevention and intervention. Hence, further comprehensive investigations are required to elucidate the precise function of ferroptosis in the pathogenesis of IBD and other associated intestinal disorders.

**Table 2:** Molecules targeting ferroptosis in IBD.

Effect	Drugs	Target	Mechanisms	Model	References
Inducers	Oral iron	Iron	Exacerbates oxidative stress through the Fenton reaction	UC rats	(78)
	AA	GPX4	Trigger epithelial LPO and an inflammatory response restricted by GPX4	CD patients, Gpx4^+/−IEC^ mice	(65)
Inhibitors	DDO7232	Nrf2	Reduces oxidative stress and decreases the inflammatory factors level	NCM460 cells, UC mice	(79)
	CsA, 6-TG	pro-inflammatory cytokines	Reduced mRNA expression of several pro-inflammatory cytokines in spleen and colon	UC mice	(80)
	Curculigoside	GPX4	Increases selenium sensitivity and promotes GPX4 expression	IEC-6 cells, UC mice	(22)
	DFO, DFP	Iron	Chelates excessive free iron and suppresses iron-dependent lipid peroxidation	UC mice	(21, 81)
	Lip-1	ROS	Lipophilic antioxidants	UC mice	(21)
	Fer-1	ROS	Blocks lipid peroxidation and restrains ROS overgeneration	UC mice	(21)
	Astragalus polysaccharide (APS)	Nrf2/HO-1	Antioxidant and anti-inflammatory properties	Caco-2 cells, UC mice	(82)

## 5 Conclusion and outlook

Despite significant advances, the examination of intestinal ferroptosis is still in its nascent phase, and its precise function in the context of IBD requires further exploration. Although we have summarized several approaches to studying ferroptosis from different aspects, there are no agreed criteria to directly define its occurrence. The identification of markers and other techniques for the assessment of ferroptosis *in vivo* is of the highest priority. Thus, biomarkers of ferroptosis may offer hope for indicating the severity of IBD. We found that the link between ferroptosis and iron/lipid peroxidation is a topic of debate. More evidence is required to establish a conclusive relationship between iron-induced atrophy, oxidative stress, and lipid peroxidation in the development and progression of the disease. Furthermore, it is imperative to investigate the signaling pathways and primary transcriptional regulators associated with iron uptake disorders in order to optimize their regulation and protect the gut against harm and carcinogenesis. Therefore, it is imperative to conduct further research on ferroptosis as a novel therapeutic target in the field of intestinal disorders.
